# Immediate implant positioning using tooth‐derived bone substitute material for alveolar ridge preservation: Preliminary results at 6 months

**DOI:** 10.1002/cre2.685

**Published:** 2022-11-10

**Authors:** Silvio Taschieri, Benedetta Morandi, Alice Alberti, Svetlana Tarasenko, Ekaterina Diachkova, Luca Francetti, Stefano Corbella

**Affiliations:** ^1^ Department of Biomedical, Surgical and Dental Sciences Università degli Studi di Milano Milan Italy; ^2^ Department of Dentistry IRCCS Istituto Ortopedico Galeazzi Milan Italy; ^3^ Department of Oral Surgery, Institute of Dentistry I. M. Sechenov First Moscow State Medical University Moscow Russian federation

**Keywords:** anterior maxilla, CBCT, dental implants, hard tissue changes, immediate implants, soft tissue changes, tooth‐derived material

## Abstract

**Objectives:**

In the present study we evaluated the effectiveness of the use of a novel autologous bone substitute derived directly from processing the extracted tooth in the upper premolar area to preserve a suitable esthetic score and functionality.

**Material and Methods:**

Fourteen bone‐level implants with platform switching were inserted in 12 patients immediately after atraumatic extraction of premolars for restorative or endodontic reasons. The implant buccal bone gap was filled using autologous tooth extracted‐derived material. Clinical and radiographic parameters, including Pink Esthetic Score (PES) were evaluated at 6 months.

**Results:**

A total of 10 patients accounting for 11 implants were included. PES showed a suitable esthetic result, and all the implant‐prosthetic rehabilitation reported full satisfaction for masticatory function, phonetics, and aesthetics. Bone levels were stable and not affected by implant location, lesion type, or bone quality.

**Conclusion:**

Radiographically the autologous bone substitute used appears stable in the surgical site and there is good continuity between the autologous bone and the graft. No adverse effects such as periodontal inflammation, infection, or graft rejection was reported.

## 1 INTRODUCTION

Implant surgery performed immediately after a tooth extraction is a long‐standing surgical practice whose functional reliability has been proven by several papers published in the scientific literature.

On the other hand, if the functionality and survival of the implants placed with this technique is proven, the same cannot be said from an esthetic point of view. In fact, in the literature we can find many protocols on how both hard and soft tissues should be managed. Several articles have presented clinical guidelines for patient selection and/or for achieving an optimal outcome for the immediate implant placement procedure, also considering esthetic outcomes (Becker, 2005; Douglass & Merin, 2002; Eini et al., 2021; Palti, 2004; Rosenquist, 1997; Schropp & Isidor, 2008; Schwartz‐Arad & Chaushu, 1997; Sclar, 2004).

Some authors indicate not to use bone substitutes or membranes but point to the physiology of wound healing as the only regenerative means (S. T. Chen et al., 2005; Paolantonio et al., 2001). Other authors, on the other hand, prefer to place bone substitutes between the implant surface and the buccal bone, using or not using a resorbable barrier membrane to cover the graft (Corbella et al., 2013). All these latter concepts are further complicated by the fact that some authors give precise indications of use based on the size of the gap between the implant and the buccal plate, while others take into account the thickness of the cortical itself and finally, when the use of the membrane is proposed, the authors propose different positioning methods. It is, therefore, very difficult to obtain reliable indications with a solid basis of scientific evidence (Feine et al., 2018).

However, given that well‐defined inclusion criteria were not yet established, an ITI Consensus delivered clear clinical guidelines in which strict inclusion criteria were emphasized as one of the main factors influencing the treatment outcome when planning and performing immediate implant placement (Feine et al., 2018).

More recently, a novel technique for obtaining an autologous bone substitute with osteoinductive and osteoconductive potential has been introduced in the dental market. This material could be derived directly from processing the extracted tooth and can be reinserted into the patient in the form of microgranules, after a specific procedure that involves the utilization of one device. The literature presents different studies indicating its positive regenerative potential and guaranteeing its safety from the microbiological point of view (Minetti, Giacometti, et al., 2020; Minetti, Taschieri, et al., 2020). Osteoinductive properties and the presence of bone morphogenetic proteins (BMPs) were demonstrated for demineralized dentin by studies published in the last decades (Bang, 1967; Bessho et al., 1991). Several studies demonstrated that the composition of hydroxyapatite and type 1 collagen was almost similar for bone, dentin, and enamel, although different proportion of essential components were detected (Y. K. Kim et al., 2014; Minetti, Giacometti, et al., 2020; Pang et al., 2017).

The tooth as a graft material has shown favorable qualities, similar to the autologous bone, as proven for the first time by Yeomans and Urist (1967). In their animal study, different tissues (tendons, muscles, decalcified and sterilized cortical bone, and dentin) where used as graft materials and analyzed up to 12 weeks: while the first two materials were replaced with fibrous tissue, the bone matrix was resorbed in 4 weeks for the bone graft, and dentin showed slower resorption and significant induction of osteogenesis. A number of different protocols have been proposed in the literature for the demineralization of the grafting material, including the use of different hydrochloric acid (HCl) solutions for 48 h, 70% ethyl alcohol, ethylenediaminetetraacetic acid (EDTA) at different pH levels, acetic acid, or nitric acid.

In this study, we wanted to evaluate the clinical effectiveness of the use of such bone substitute material of autogenous origins applied in the post‐extraction implantology technique in the premolar area of the upper jaw to preserve a suitable esthetic score and functionality, using an automated device.

## 2 MATERIALS AND METHODS

This clinical study was based on a series of patients treated at the private office in Milano, Italy, between February 2018 and June 2019. All patients were treated following the principles embedded in the Helsinki Declaration (Galeazzi Institute Hospital approved by institutional review board ‐ L231). One experienced surgeon (ST) performed the operations. A total of 12 patients received a total of 14 implants, inserted immediately after tooth extraction at the anterior maxilla, in the premolar area. All patients were recalled at six months follow‐up for clinical and radiographic evaluation.

### 2.1 Inclusion criteria

Patients with more than 18 years, in good general health with no controindications for oral surgical procedures (ASA‐1 or ASA‐2 according to the classification of the American Society of Anesthesiologist) and with healthy periodontium, demonstrating the need for immediate implant replacement (Blanco et al., 2019) in the premolar area of the upper jaw after the extraction of one tooth due to endodontic/restorative reasons. All patients were nonsmokers or former smokers or smoke less than 10 cigarettes a day and were able to understand the study procedure and to understand and sign an informed consent.

### 2.2 Exclusion criteria

Patients with ongoing periodontal disease and bone loss or/and with the inability to maintain reasonable oral hygiene; patients with history of alcohol, narcotics or drug abuse; patients with a history of radiotherapy in the head and neck region or currently under chemotherapy; patients with parafunctional habits (severe to moderate Bruxism) or/and temporomandibular joint disease; patients with a site where a history of failed implant exists.

### 2.3 Surgical procedure

One hour before surgery, patients took 2 g of amoxicillin and clavulanic acid (Augmentin; Roche, Milan, Italy) for antibiotic prophylaxis (Esposito, Grusovin, Loli, et al., 2010; Hammerle et al., 2004). Local anesthesia was obtained with articaine chlorohydrate (4%) and epinephrine (1:100,000; Alfacaina N; Weimer Pharma, Rastat, Germany).

The first step of the surgical procedure consisted of a careful tooth extraction with the intention of minimizing the mechanical trauma of the surrounding bone. Then, the socket was thoroughly debrided. Bone‐level implants, with platform switching (V3; MIS Implant Technologies Ltd., Bar‐Lev Industrial Park, Israel or Neo implant Alpha‐Bio Tec, Kiryat Arye, Petach Tikva, Israel), were immediately placed in the prepared sites. Implant site preparation was performed according to a correct prosthetic positioning and its apical portion was under‐prepared to increase the possibility of obtaining implant primary stability. Care was taken to place the implant shoulder approximately 3–4 mm below the ideal buccal gingival margin of the future restoration. The implant buccal bone gap was filled using autologous tooth extracted‐derived material in the same surgical procedure (Tooth Transformer®; SRL, Via Washington, Milan, Italy) in all cases.

The material was placed in the gap between the buccal bone and the implant where the space width was equal to or greater than 2 mm. If the gap was less than 2 mm, the material was grafted both in the small residual space remaining between the surface of the implant and the buccal bone, and between the buccal bone and the periosteum, after atraumatic elevation of a muco‐periosteal flap, not involving the interdental papillae.

The procedure to use autologous extracted teeth as bone substitute material was as follows: the selected teeth were completely cleaned of any residues of calculus, soft tissues, and restoration materials. Then, they were cut into small portions; during this phase, endodontic filling material were also removed easily by direct visualization. The so‐obtained fragments were grinded using a low‐speed grinder and demineralized and sterilized through a standardized and automatic procedure using a specific device (Tooth Transformer®; SRL). During the automatic process, the granules undergo a treatment with different liquids (0.1 M HCl, 10% hydrogen peroxide [H_2_O_2_], demineralized water), UVA rays and ultrasonic vibrations, with a temperature <43°C to avoid protein denaturation, thus preserving the protein content. The resulting biomaterial, made of both dentin and enamel granules (diameter between 0.4 and 0.8 mm) was placed in the surgical site.

Soft tissues were sutured with bioresorbable material (Vicryl 5‐0; Ethicon Inc., Johnson & Johnson, Bridgewater).

At the end of the surgical procedure, patients were instructed to rinse their mouth twice a day for 1 week with 0.2% chlorhexidine digluconate mouthwash and to take anti‐inflammatory drugs for pain control (nimesulide 100 mg twice a day). A soft diet was prescribed for a few days in order to avoid mechanical contact of food with the surgical site. Sutures were removed after 7 days from surgery.

Immediate loading with provisional abutments and resin crowns was delivered for all cases in the subsequent 24 h, given there was an implant insertion torque value between 30 and 45 Ncm. Definitive restorations in zirconia were delivered after a minimum of 4 months healing time before the first removal (disconnection) of the provisional restoration.

### 2.4 Clinical examination

Before surgery, the following parameters were evaluated:

Clinical parameters: Gingival biotype (GB) measured by means of periodontal probe assessment (as described by De Rouck et al., 2009), which was shown to be significantly superior to visual measurements and not significantly different to direct measurements, (Kan et al., 2010) width of keratinized mucosa (KM); values for KM, FTCH and CTCH were measured using a periodontal probe to the nearest millimeter.

### 2.5 Radiographic examination

A periapical radiograph was taken during the first visit. A CBCT was taken with the same device 3D Accuitomo XYZ Slice View Tomograph® (Model MCT‐1, Type EX‐1/EX‐2; J. Morita Mfg. Corp, Fushimi‐ku, Kyoto, Japan) with 60–80 kV and 1–10 mA, a voxel size of 0.125 mm per side, and an approximate exposure time of 18 s. before tooth extraction. CBCTs examination was performed in order to be able to adequately plan the surgical act, to visualize the position of the maxillary sinus and other anatomical structures, observe the residual bone at the apical level, and note the presence of buccal bone and identify the correct size, length, and position of the implant.

Immediately after implant insertion and after 6 months, a periapical radiograph was taken.

Intraoral periapical radiographs were taken using a long cone paralleling technique. In order to ensure reproducibility, individual trays were used. Each radiograph was scanned at 600 dpi (EpsonPerfection Pro; Epson, Suwa, Japan). Marginal bone levels around implants were measured using a dedicated image analysis software (UTHSCSA Image Tool, version 3.00 for Windows; University of Texas Health Science Center, San Antonio, TX) using the implant neck as a reference. Both mesial and distal aspect of the implant were assessed; the mean value between the mesial and distal measurements was calculated.

## 3 OUTCOME VARIABLES

The outcome variables were evaluated 6 months after implant placement:

1. *Implant survival*, evaluated based on the following criteria: presence of the implant in the patient's mouth, absence of peri‐implant radiolucency, no recurrence or persistent peri‐implant infection and no complain of pain and of neuropathies or paresthesia.
2. *Prosthesis success*, defined by the presence of functional prosthesis in patient's mouth without mechanical complications.
3. *Peri‐implant bone loss*.
4. *Pink Esthetic Score (PES)* (Fürhauser et al., 2005).

## 4 PES INDEX

In order to evaluate objectively the esthetic outcome of the implant crowns at each of the scheduled stage, the PES index (18) was scored by a single operator and critically controlled by two independent expert examiners (ST, LF), with more than 10 years implant clinical practice, via digital images. In case of disagreement, a third expert clinician (SC) evaluated the images, and the value was assigned after all three clinicians had reached a common rating. The PES index consisted of five parameters with a maximum score of 10, representing optimum esthetic outcome with respect to the peri‐implant soft tissue conditions.

## 5 RESULTS

Two patients were excluded as they were not present at the 6‐month follow‐up visit. A total of 10 patients accounting for 11 implants (four maxillary first premolars and seven maxillary second premolars) were evaluated.

PES index scores at 6 months follow‐up showed a suitable esthetic result showing a mean score of 8.

**Figure 1:**
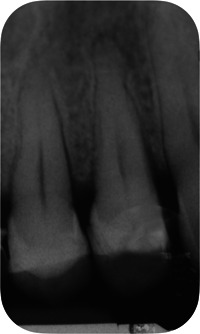
Baseline radiograph of the hopeless tooth

**Figure 2:**
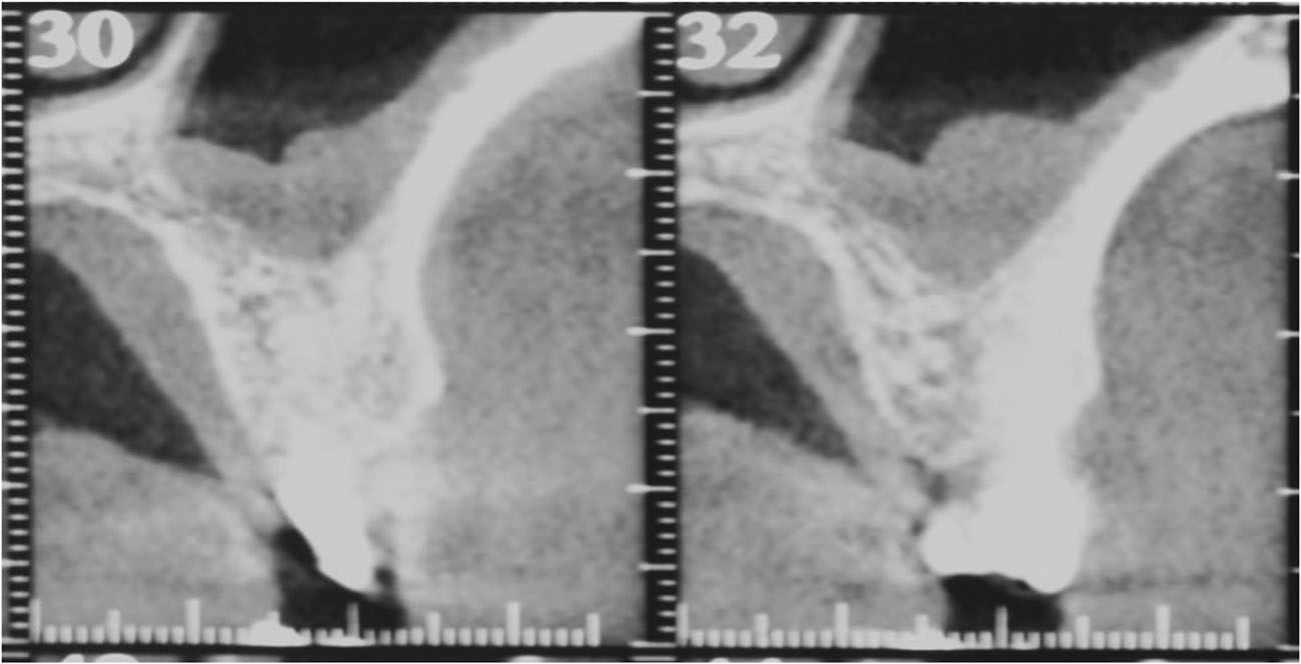
Presurgical cone beam computed tomography

**Figure 3:**
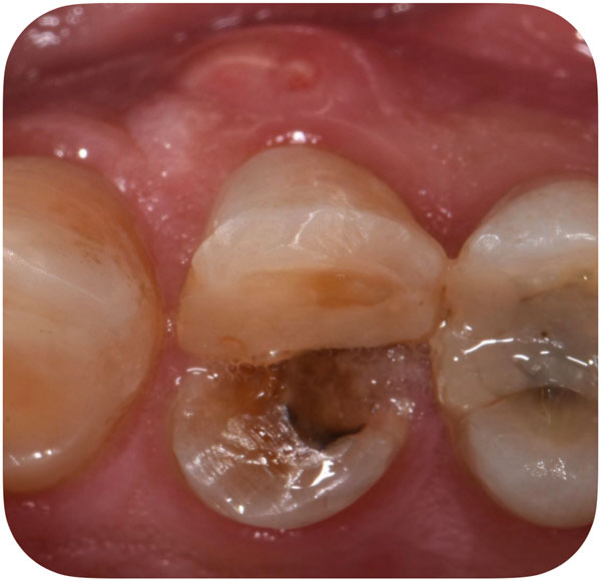
Clinical situation of the hopeless tooth, showing a vertical root fracture

**Figure 4:**
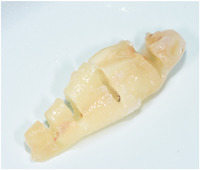
Tooth cleaned and prepared for processing

**Figure 5:**
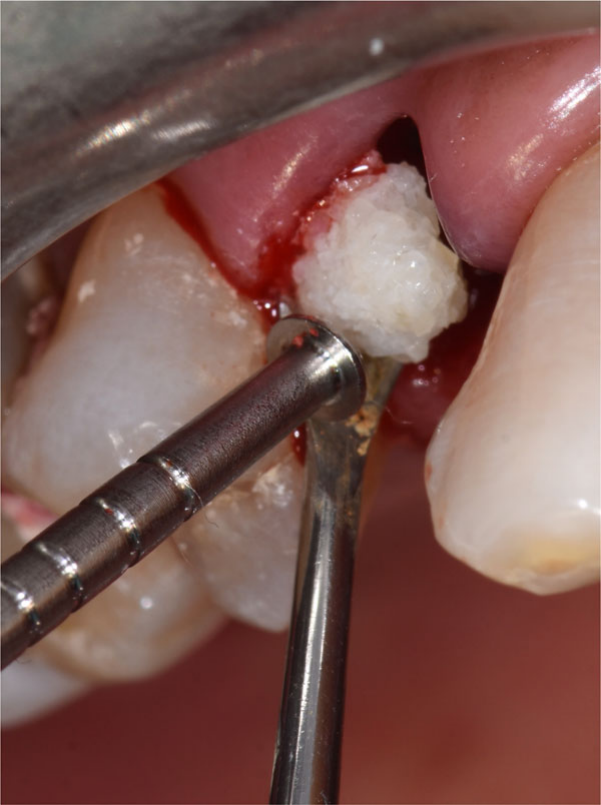
Positioning of the autologous tooth‐derived graft material in the implant‐buccal bone gap

**Figure 6:**
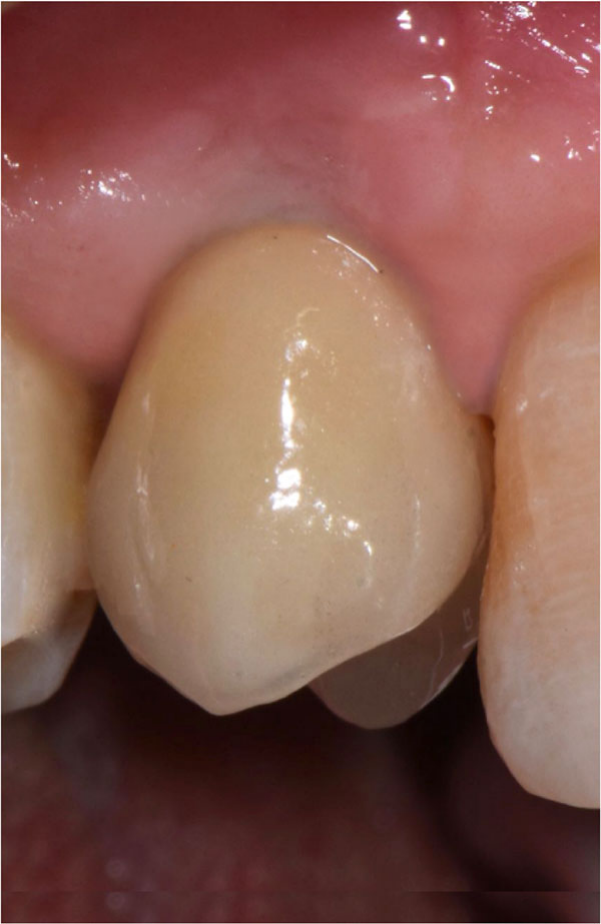
Clinical evaluation of the implant‐supported prosthesis at the 6‐month follow‐up visit

**Figure 7:**
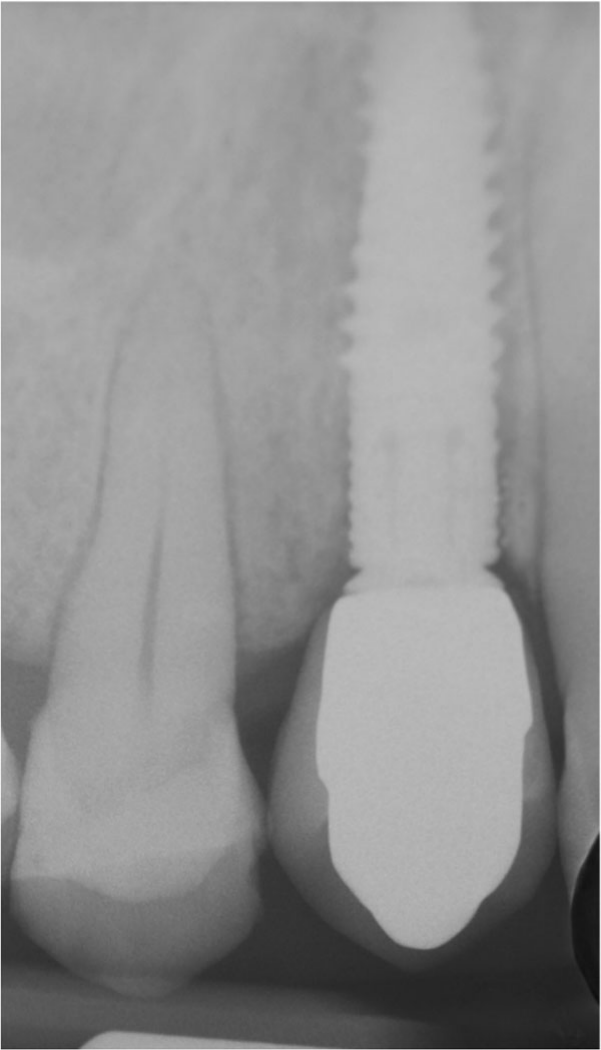
Radiographic evaluation of the postextractive implant at 6 months

Bidimensional measurements of bone resorption parameters at 6 months follow‐up were: mesial 0.39 ± 1.19 mm; distal 0.42 ± 0.90 mm. Such a value was not affected by implant location (first or second premolar), lesion type, or bone quality (Q2 or Q3). A clinical case is shown in Figures 1, 2, 3, 4, 5, 6, 7 as an example. All implant‐prosthetic rehabilitation evaluated reported full satisfaction for mastication function, phonetics, and esthetics.

## 6 DISCUSSION

When deciding to rehabilitate a post‐extraction site, the operator can choose between at least three different protocols regarding the timing of implant insertion in relation to the time of extraction: immediate placement, immediate‐delayed placement after soft tissue healing, and delayed placement, which includes the complete bone healing (Esposito, Grusovin, Polyzos, et al., 2010).

Actually, immediate implant placement constitutes a treatment of choice in cases showing a good preservation of the socket's walls after the extraction (Becker, 2005). Nevertheless, physiological alterations of buccal and palatal/lingual bone surfaces should be taken into consideration and evaluated, so that this approach may be considered at risk for esthetic reasons (Buser et al., 2017).

Most of the studies agreed that there is a higher risk of mucosal recession, in the range of 20%–30%, related to immediate implant placement when compared to other protocols, in which implants are placed after bone healing (S. Chen & Buser, 2014). For this reason, in a specific clinical condition such as in the esthetic area, soft tissue augmentation procedures could be indicated (Thoma et al., 2021).

However, one of the main advantages of immediate implant placement is that it permits to significantly decrease the rehabilitation time for the patients. On the other hand, providing an initial provisional restoration immediately after implant insertion, introduces possible advantages in terms of reducing bone contour changes (Tarnow et al., 2014) and achieving an immediate adequate esthetic outcome.

In this clinical study, an attempt was made to acknowledge all possible biological advantages for the benefit of the patient. The techniques of immediate implant placement together with immediate loading were supplemented using an autologous graft material.

The use of tooth‐derived grafting material following the technique described above have been proposed recently and it has gained increasing interest as showed in various studies (Del Canto‐Diaz et al., 2019; Dental et al., 2019; S. Y. Kim et al., 2017; Minamizato et al., 2018; Pang et al., 2017). In a prospective clinical trial including 33 graft sites, Pang et al. (2017) showed a similar result on bone regeneration using autogenous tooth graft material compared with an organic bovine bone.

This can be explained because the autogenous demineralized dentin matrix (ADDM) represents an efficient carrier of different bioactive growth factors (GFs), such as BMPs and transforming growth factor‐β (TGF‐β). Actually, when BMPs are used alone, they cannot show osteoinductive effects due to their high solubility. Such GFs that have been proved to be present in the dentin are involved in the repairing process of the bone, thus potentially enhancing the bone healing processes Nakashima (2005).

In order to preserve the above‐mentioned organic autologous components, it is fundamental to follow a strict preparation technique. Moreover, it is of utmost importance to remove any contaminants to avoid inflammatory or infective reactions and prepare the inorganic part to be easily colonized by osteoblasts. The demineralization process is a key step to spread out GFs and proteins because hydroxyapatite crystals have reported to inhibit their release from the tooth matrix (Bessho et al., 1991; Blum et al., 2004; Y. K. Kim et al., 2013).

A key point of the present study is the use of a standardized protocol for all cases. As stated before, a standardization of the heterogeneous protocols for tooth demineralization proposed in the scientific literature is needed. In our study, the use of a specific device guarantees the uniformity of the process. Moreover, it gives assurance of the maintenance of an adequate protein content. A residual weight of at least 3.5%–4% of the tooth should be reached at the end of the process, to have enough proteins, thus allowing the graft to exhibit osteoinductive properties, in addition to osteoconduction (Minetti, 2021).

In 2016, a case series was published showing that the cortico‐cancellous bone that had formed using autogenous tooth bone graft material had been maintained successfully with an average follow‐up of 5 years. In this study, CBCT was used to measure the width and the height of the graft and histological analyses have been taken to demonstrate cortico‐cancellous bone formation (Y. K. Kim et al., 2016).

In 2006, other authors (Gomes et al., 2006) applied ADDM in the third molar socket and observed through radiographic analysis that this material gradually disappeared after 90 days showing a better bone architecture and a faster repair process than a polytetrafluoroethylene barrier and a dental socket alone. Their findings imply the resorption of ADDM during the healing process.

Our preliminary results confirmed that this grafting technique can be applied with satisfactory esthetic outcomes and a suitable functionality.

One limitation of the study, together with the relatively limited number of patients, is the short‐term assessment of the peri‐implant bone level, only 6 months after implant placement. In the future, a larger number of patients together with a longer follow‐up will be necessary parameters for a better evaluation of the technique proposed together with a randomized study design. Moreover, it should be pointed out that all patients recruited in this study had a medium/thick biotype and therefore the possibility of achieving good esthetic parameters was probably easier. It will be interesting in further studies to perform this surgical/prosthetic technique also in patients with a thin biotype.

In this clinical study, there were no adverse effects such as periodontal or bone tissue inflammation, infection, or graft rejection. The actual metabolic process at the surgical site was not detected due to the ethical impossibility of taking histological samples and analyzing them. Radiographically, the autologous bone substitute used appears stable in the surgical site and there is good continuity between the autologous bone and the graft.

## AUTHOR CONTRIBUTIONS

**Silvio Taschieri**: conceptualization, project administration, data collection, supervision, validation. **Benedetta Morandi**: writing–original draft, review and editing, investigation, validation. **Alice Alberti**: writing–original draft, review and editing, investigation, validation. **Svetlana Taschieri**: data curation, review and editing, validation. **Ekaterina Diachkova**: data curation, review and editing, validation. **Luca Francetti**: supervision, methodology, validation. **Stefano Corbella**: supervision, methodology, project administration, validation. All authors have read and agreed to the published version of the manuscript.

## CONFLICT OF INTEREST

The authors declare no conflict of interest.

## Data Availability

Data available on request from the authors.
